# Lithium-Decorated Borospherene B_40_: A Promising Hydrogen Storage Medium

**DOI:** 10.1038/srep35518

**Published:** 2016-10-18

**Authors:** Hui Bai, Bing Bai, Lin Zhang, Wei Huang, Yue-Wen Mu, Hua-Jin Zhai, Si-Dian Li

**Affiliations:** 1 Key Laboratory of Coal Science and Technology of Ministry of Education and Shanxi Province, Taiyuan University of Technology, Taiyuan 030024, Shanxi, China; 2 Nanocluster Laboratory, Institute of Molecular Science, Shanxi University, Taiyuan 030006, Shanxi, China; 3 State Key Laboratory of Quantum Optics and Quantum Optics Devices, Shanxi University, Taiyuan 030006, Shanxi, China

## Abstract

The recent discovery of borospherene B_40_ marks the onset of a new kind of boron-based nanostructures akin to the C_60_ buckyball, offering opportunities to explore materials applications of nanoboron. Here we report on the feasibility of Li-decorated B_40_ for hydrogen storage using the DFT calculations. The B_40_ cluster has an overall shape of cube-like cage with six hexagonal and heptagonal holes and eight close-packing B_6_ triangles. Our computational data show that Li_*m*_&B_40_(**1**–**3**) complexes bound up to three H_2_ molecules per Li site with an adsorption energy (AE) of 0.11–0.25 eV/H_2_, ideal for reversible hydrogen storage and release. The bonding features charge transfer from Li to B_40_. The first 18 H_2_ in Li_6_&B_40_(**3**) possess an AE of 0.11–0.18 eV, corresponding to a gravimetric density of 7.1 wt%. The eight triangular B_6_ corners are shown as well to be good sites for Li-decoration and H_2_ adsorption. In a desirable case of Li_14_&B_40_-42 H_2_(**8**), a total of 42 H_2_ molecules are adsorbed with an AE of 0.32 eV/H_2_ for the first 14 H_2_ and 0.12 eV/H_2_ for the third 14 H_2_. A maximum gravimetric density of 13.8 wt% is achieved in **8**. The Li-B_40_-*n*H_2_ system differs markedly from the previous Li-C_60_-*n*H_2_ and Ti-B_40_-*n*H_2_ complexes.

Due to its merits of cleanness, renewability, abundance in nature, and high energy density per unit mass, hydrogen has been recognized as an appealing energy carrier for the future world. It has the potential to reduce our dependence on fossil fuels, which are limited in resource and harmful to the environment^1^,^2^,^3^,^4^. One bottleneck of using hydrogen for vehicular applications is the lack of safe and efficient hydrogen storage materials^5^,^6^,^7^ that store molecular H_2_ reversibly with high gravimetric density and fast kinetics for adsorption, as well as desorption, under the conditions of moderate temperature and pressure^8^,^9^. An ideal H_2_ storage system would be one that binds hydrogen in molecular form and with an adsorption energy (AE) in the regime of 0.1–0.5 eV per H_2_, that is, intermediate between the physisorbed and chemisorbed states^10^,^11^. Although advances have been made towards meeting the U.S. DOE’s targets for hydrogen storage, an ideal system is yet to be designed and synthesized. Therefore, seeking novel hydrogen storage materials has remained an important issue.

Previous experiments and theoretical calculations have shown that metal-decorated carbon fullerenes and nanotubes^12^,^13^,^14^,^15^,^16^,^17^,^18^,^19^, as well as their boron-, nitrogen- and beryllium-substituted nanostructures^20^,^21^,^22^, might be good candidates for the storage of H_2_ molecules. For instance, Zhang and co-workers showed that the reversible hydrogen storage of transition-metal-coated C_60_ and C_48_B_12_ may be as high as 9 wt%^21^. Yildirim *et al*. revealed that Ti-coated single-walled carbon nanotubes can store 8 wt% of H_2_^23^. To avoid the clustering problem of transition metal atoms on the surface of carbon nanostructures, Yoon and co-workers^18^ found that Ca can achieve homogeneous monolayer coating, which is superior to other metal elements. They concluded that up to 8.4 wt% of hydrogen can be stored in Ca_32_C_60_ with an AE of 0.2–0.4 eV/H_2_. Through first-principles computations, Sun *et al*.^13^ predicted that Li-decorated fullerene C_60_ (Li_12_C_60_) can store up to 9 wt% of H_2_, albeit with a rather weak AE of 0.075 eV/H_2_. Furthermore, Yoshida *et al*.^17^ measured the hydrogen absorption of Li_9_C_60_ based on experiments and confirmed that up to ~2.6 wt % H_2_ can be stored at 250 °C and 30 bar H_2_. For lithium-doped fullerenes (Li_x_-C_60_-H_y_) with a Li:C_60_ mole ratio of 6:1, a reversible uptake of 5 wt% H_2_ at 350 °C and 105 bar H_2_ and desorption onset temperature of ~270 °C was observed^15^. Subsequently, another experimental results^16^ showed that up to 9.5 wt % deuterium (D_2_) are absorbed in Li_12_C_60_ under a pressure of 190 bar and a temperature below 100 °C.

Boron is the lighter neighbor of carbon in the periodic table, which possesses the similar merit as carbon in terms of light weight and potential applications for hydrogen storage. For this purpose, its chemical hydrides^24^,^25^,^26^ were studied, as were relevant model nanostructures, such as boron monolayer sheets, fullerenes, and nanotubes^27^,^28^,^29^. In particular, following the proposal of the celebrated *I*_*h*_ B_80_ buckyball^30^, which is built upon the C_60_ motif by capping all 20 surface hexagons, a number of papers were devoted to hydrogen storage using B_80_ coated with metals (M = Li, Na, K, Be, Mg, Ca, Sc, Ti, and V)^27^,^31^,^32^,^33^. However, B_80_ was subsequently found to favor core-shell type structures at various theoretical levels^34^,^35^. It is thus not feasible to pursue any realistic technological applications of B_80_ buckyball as hydrogen storage materials.

Very recently, the first all-boron fullerenes or borospherenes, *D*_2*d*_ B_40_ and *D*_2*d*_ B_40_^−^, were observed in a combined experimental and theoretical study^36^, marking the onset of the borospherene chemistry, whose future development may be envisioned to parallel that of the fullerenes. Endohedral M@B_40_ (M = Ca, Sr) and exohedral M&B_40_ (M = Be, Mg) metalloborospherenes were also predicted, which further support the structural, electronic, and chemical robustness of the B_40_ borospherene^37^. Closely following B_40_, chiral B_39_^−^, B_41_^+^, and B_42_^2+^ borospherenes were also studied^38^,^39^, which expand the borospherene family and may eventually lead to new boron-based nanomaterials.

Borospherene B_40_ possesses a cube-like cage structure, whose six hexagonal and heptagonal holes each occupy a face of the cube. It also has eight triangular, close-packing B_6_ structural blocks, each on an apex of the cube. All B atoms are on the surface of the cage, which is an ideal, well-defined system for chemistry. B_40_ differs from carbon fullerenes in terms of structure and bonding, and the pursuit of borospherene-based nanomaterials for hydrogen storage is thus intriguing from a fundamental point-of-view. Furthermore, borospherenes are lighter than carbon fullerenes, which make the former systems better candidates to reach a higher gravimetric capacity for hydrogen storage. Relevant to this topic, Dong *et al*.^40^ predicted on the basis of density-functional theory (DFT) calculations that Ti-decorated B_40_ fullerene (Ti_6_B_40_) is capable of storing up to 34 H_2_ molecules with a maximum gravimetric density of 8.7 wt% and a reversible storage capacity of 6.1 wt%. To our knowledge, the U.S. DOE has set a target of 7.5 wt% for hydrogen storage capacity for the year of 2015^41^,^42^.

In this work, we choose to study lithium-decorated borospherene B_40_ as a potential candidate for hydrogen storage via extensive DFT calculations. Since boron-based nanomaterials are also candidates for lithium storage, the current ternary B-Li-H system is quite unique^28^,^29^,^43^,^44^. Compared to transition metal, Li as the lightest metal definitely will facilitate the improvement of hydrogen storage capacity for the metal-decorated B_40_ system. The Li_*m*_-B_40_-*n*H_2_ system differs markedly from Li_*m*_-C_60_-*n*H_2_ or Ti_6_-B_40_-*n*H_2_, which have an AE value that is either rather small (0.075 eV)^13^ or too large (up to 0.82 eV)^40^. Even the recently proposed Li_8_-B_6_-*n*H_2_ system^44^ only has an AE of less than 0.1 eV. Our computational data show that Li-decorated B_40_ appears to be a promising medium for hydrogen storage. The Li atoms readily attach to the top of hexagonal and heptagonal holes on B_40_, forming a series of charge-transfer complexes from *C*_*s*_ Li&B_40_(**1**), *C*_2*v*_ Li&B_40_(**2**), up to *D*_2*d*_ Li_6_&B_40_(**3**). The Li_*m*_&B_40_ complexes can adsorb three H_2_ molecules per Li site with a moderate AE of 0.11–0.25 eV/H_2_. The Li_6_&B_40_(**3**) complex stores up to 34 H_2_ with an average AE of 0.10 eV/H_2_. The first 18 H_2_ of these possess ideal AEs, which suggest a gravimetric density of 7.1 wt%. Furthermore, the eight close-packing, triangular B_6_ corner sites of B_40_ are also suitable for Li-decoration and H_2_ adsorption. In a desirable Li_14_&B_40_(**7**) complex, up to 42 H_2_ molecules can be stored with AEs of 0.12–0.32 eV/H_2_, which corresponds to a gravimetric density of 13.8 wt%.

## Results and Discussion

### Isolated B_40_ Borospherene for H_2_ Adsorption

The first all-boron fullerene called as borospherene^36^, D_2d_ B_40_ (^1^A_1_), possesses a very large HOMO–LUMO gap of 3.13 eV at the PBE0 level that indicates its overwhelming stability, which is comparable to that of I_h_ C_60_ (^1^A_g_) (3.02 eV) calculated at the same level. All the valence electrons in B_40_ are either delocalized σ or π bonds and there is no localized 2c–2e bond, unlike the C_60_ fullerene. In fact, the surface of B_40_ is not perfectly smooth and exhibits unusual heptagonal faces which may play a role that release the surface strains, in contrast to C_60_ fullerene whose surface makes up of pentagons and hexagons and presents the least strain. And the diameter of B_40_ is 6.2 Å, slightly smaller than the value of C_60_ (7.1 Å), which makes B_40_ more comfortable to accommodate a range of small molecules inside the cage.

**Figure 1:**
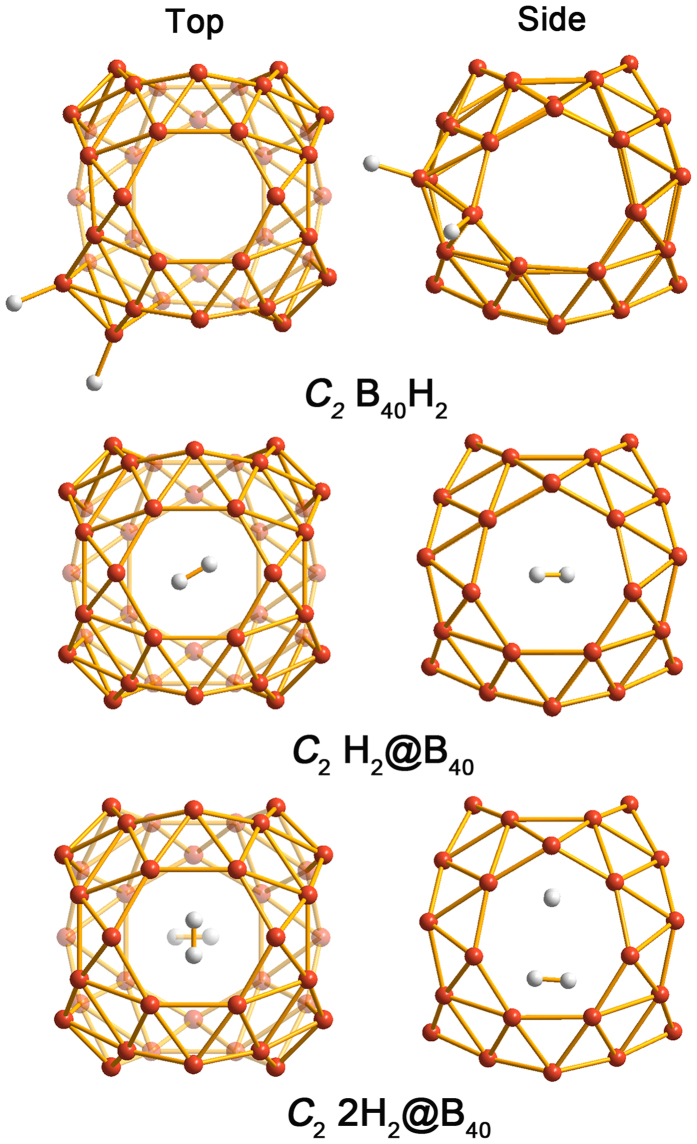
Top and side views of optimized configurations of B_40_H_2_, H_2_@B_40_ and 2H_2_@B_40_.

We initially studied H_2_ adsorption on the isolated B_40_ borospherene. The optimized structures of B_40_H_2_, H_2_@B_40_ and 2H_2_@B_40_ are shown in Fig. 1. In the *C*_2_ B_40_H_2_ dihydride, the H_2_ molecule tends to form two B–H covalent bonds with the tetracoordinate-B sites, which dissociate H_2_. The dissociative AE of a single H_2_ is up to 1.30 eV. For H_2_ storage inside the cage, only one H_2_ molecule can be encapsulated into B_40_, which is marginally exothermal with an AE of 0.24 eV. Interestingly, once such an encapsulation is completed, the H_2_ molecule cannot escape due to substantial energy barriers (>3 eV). The AE of a second H_2_ inside the cage is found to be endothermic by 1.32 eV, which is thus not feasible experimentally. In short, the above results show that an isolated B_40_ borospherene is not a good candidate for hydrogen storage directly. The B_40_-H_2_ interactions appear to be different from the case of C_60_. The latter is known to interact with H_2_ via weak van der Waals forces^45^. As a comparison, our calculation results show that the dissociative AE of a single H_2_ for C_60_H_2_ is only ~0.18 eV at the same level. However, similar to B_40_, only one hydrogen molecule can reside inside the C_60_ cage with a negative AE value of ~0.22 eV.

### Configurations of Li-Decorated B_40_

**Figure 2:**
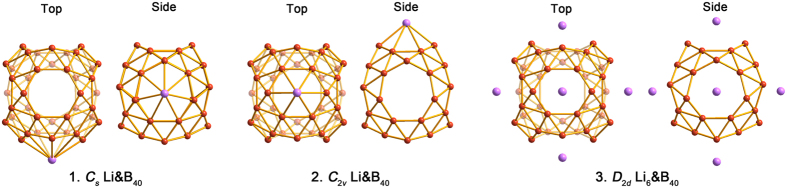
Top and side views of the optimized configurations of *C*_*s*_ Li&B_40_(**1**), *C*_2*v*_ Li&B_40_(**2**), and *D*_2*d*_ Li_6_&B_40_(**3**), in which one Li atom is coated on a heptagonal hole of B_40_, one Li atom is on a hexagonal hole, and six Li atoms are on the heptagonal/hexagonal holes, respectively. The B atom is in orange and Li is in purple.

As shown in Fig. 2, we start with a single Li atom interacting with B_40_. Relative stability of Li atom bound on heptagonal and hexagonal holes were considered. The exohedral *C*_*s*_ Li&B_40_(**1**), in which Li caps a heptagon, turns out to be more stable by 0.20 eV with respect to *C*_2*v*_ Li&B_40_(**2**). In the latter species, Li caps a hexagon. The BE for Li is 3.08 and 2.88 eV in **1** and **2**, respectively. Thus, Li prefers to bind on top of the heptagonal hole of B_40_. The Li–B distance in **1** is 2.34 Å, compared to 2.33 Å in **2** (Table 1). Clearly, the BE of Li on the center of heptagon or hexagon in B_40_ is substantially higher than those on the pentagon of C_60_ (1.80 eV), in Li_2_ dimer (0.95 eV), and in the Li bulk (1.63 eV)^13^. This should help suppress the potency of Li aggregation to form clusters on B_40_ surface, suggesting that Li is a suitable adsorbate to decorate B_40_. As shown in Table 1, electron transfer occurs from Li to borospherene B_40_ cage in **1** and **2**, resulting in a positive charge of 0.87–0.88 |e| on Li as revealed in the Bader charge analysis. The ionized Li atom hints a possibility for H_2_ adsorption via the polarization mechanism^18^.

To increase the coverage of Li on B_40_, we place one Li atom on top of every hexagon and heptagon hole and reach exohedral *D*_2*d*_ Li_6_&B_40_(**3**) (Fig. 2). In complex **3**, six Li atoms remain isolated on the surface holes, resulting in a highly symmetric *D*_2*d*_ geometry. The average BE of Li in **3** is 3.07 eV/Li, which is comparable to that in Li&B_40_(**1**) (3.08 eV) and is slightly larger than that in Li&B_40_(**2**) (2.88 eV). There appears to be a collective effect for Li adsorption because six Li atoms in **3** have a higher total BE (18.48 eV) than six individual Li atoms in **1** and **2** combined (18.08 eV). This fact suggests that Li_6_&B_40_(**3**) is a favorable configuration for Li-decoration. Remarkably but not surprisingly, our computational data indicate that **3** is at least 6.29 eV more stable than B_40_ attached by a compact Li_6_ cluster (Fig. S1). Therefore, surface aggregation of Li for island clusters is unlikely in the Li_6_&B_40_ system. The Li–B distance in **3** is 2.33 Å, nearly identical to those in **1** and **2**. Bader charge analysis shows that the atomic charge on each Li atom in **3** is +0.87 |e|.

**Figure 3:**
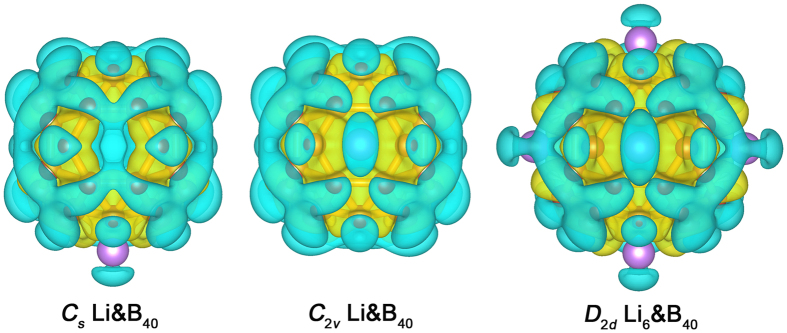
Top views of the isosurface of charge density differences of *C*_*s*_ Li&B_40_(1), *C*_2*v*_ Li&B_40_(2), and *D*_2*d*_ Li_6_&B_40_(3). Yellow color represents the electron accumulation region, and blue represents the electron depletion region.

We further analyzed the isosurfaces of charge density differences in complexes *C*_*s*_ Li&B_40_(**1**), *C*_2*v*_ Li&B_40_(**2**), and *D*_2*d*_ Li_6_&B_40_(**3**), as depicted in Fig. 3. Here the yellow and blue colors represent electron accumulation and depletion regions, respectively. From the charge density variations of **1**–**3**, which are induced by the adsorption of Li atoms onto B_40_, it is obvious that charge transfer from Li atom to B_40_ indeed takes place upon Li decoration.

**Figure 4:**
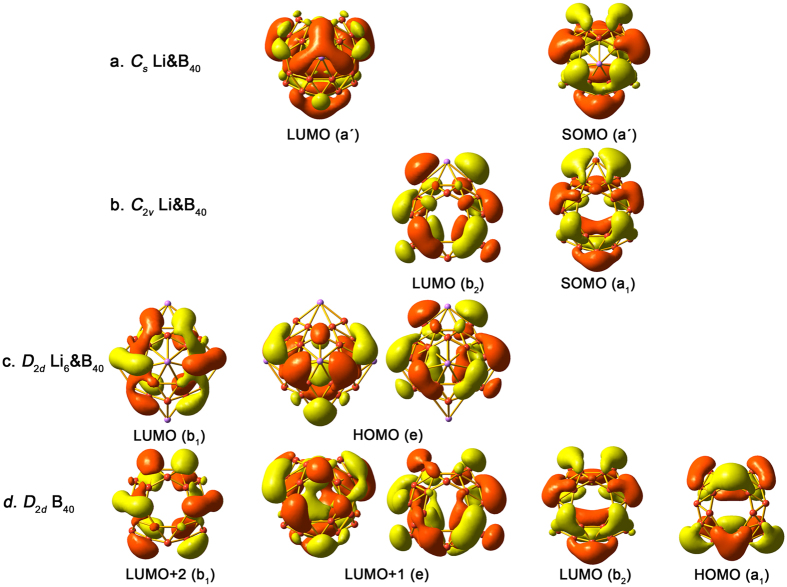
Molecular orbital pictures of the LUMOs and HOMOs (or SOMOs) of *C*_*s*_ Li&B_40_(1), *C*_2*v*_ Li&B_40_(2), and *D*_2*d*_ Li_6_&B_40_(3), as compared with those of borospherene *D*_2*d*_ B_40_. The orbitals are aligned according to their shapes.

Figure 4 shows the frontier canonical molecular orbitals (CMOs) of **1**–**3**, which are compared with those of *D*_2*d*_ B_40_. Upon attachment of the first Li atom on B_40_, the lowest unoccupied molecular orbital (LUMO) of B_40_ (Fig. 4d) becomes half-filled due to charge transfer, which are the singly occupied molecular orbitals (SOMOs) in **1** and **2** (Fig. 4a,b). Note these three CMOs are virtually identical. Likewise, in line with their lower symmetry, LUMO (a′) of **1** and LUMO (b_2_) of **2** correspond to the degenerate LUMO + 1 of *D*_2*d*_ B_40_. In *D*_2*d*_ Li_6_&B_40_(**3**) (Fig. 4c), six electrons are transferred from Li to the B_40_ cage, which successively occupy the LUMO and degenerate LUMO + 1 of *D*_2*d*_ B_40_. The latter LUMO + 1 become the highest occupied molecular orbital (HOMO) in **3**, which are also doubly degenerated due to the same high symmetry of *D*_2*d*_. As a consequence, the LUMO + 2 (b_1_) of *D*_2*d*_ B_40_ becomes the LUMO in **3**. The spatial distributions of the frontier CMOs in these four species are remarkably similar, demonstrating the electronic robustness of the B_40_ cage motif along this series. The calculated Wiberg bond indices associated with Li are all less than 0.30 in **1**–**3**, which further indicate that Li does not participate in the covalent bonding of B_40_.

The calculated HOMO-LUMO energy gaps of **1**, **2**, and **3** are 1.39, 1.41, and 1.42 eV, respectively, which differ from that of *D*_2*d*_ B_40_ (3.13 eV at the same level), suggesting the possibility to tune the electronic properties of borospherenes via metal-decoration, similar to the case of C_60_ buckyball.

### H_2_ Adsorption on Li-Decorated B_40_

The *C*_*s*_ Li&B_40_(**1**), *C*_2*v*_ Li&B_40_(**2**), and *D*_2*d*_ Li_6_&B_40_(**3**) clusters are well-defined molecular systems for the adsorption of H_2_ molecules. H_2_ can be added successively to the systems, from one H_2_ molecule up to six per Li site. The largest complex corresponds to the adsorption of 34 H_2_ molecules to **3**, that is, Li_6_&B_40_(**3**)-34 H_2_. Tables S1 and 2 summarize the AEs and the equilibrium Li–B, Li–H, and H–H distances for the Li_*m*_-B_40_-*n*H_2_ (*m* = 1, 6; *n* = 1–34) complexes.

When one H_2_ molecule is introduced to **1**, due to the polarization interaction between the charged Li atom and the H_2_ molecule, the Li–B bond distance is slightly enlarged (by 0.01 Å) to 2.35 Å. The H–H distance is found to be 0.76 Å. The equilibrium Li–H distance is 1.97 Å, which is comparable to the value of 2.04 Å in the case of a free Li^+^ ion interacting with H_2_^46^. The AE of the first H_2_ to **1** is 0.25 eV, which is in quantitative agreement with that in Li^+^H_2_ (0.25 eV)^46^.

**Figure 5:**
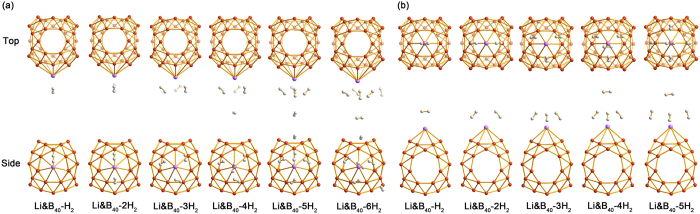
Top and side views of the optimized configurations for successive addition of H_2_ molecules on (**a**) Li&B_40_(**1**) with a Li-coated heptagonal hole and (**b**) Li&B_40_(**2**) with a Li-coated hexagonal hole.

With more H_2_ molecules being attached to **1**, the average AE of H_2_, consecutive AE of H_2_, the Li–B distance, and the distances between H_2_ and Li change accordingly. As shown in Table S1 and Fig. 5a, a single Li atom in **1**, coated on a heptagonal hole, can adsorb up to six H_2_ molecules with an average AE of 0.11 eV/H_2_. From one to six H_2_, the average AE decreases from 0.25 to 0.11 eV/H_2_, whereas the consecutive AE decrease from 0.25 to 0.05 eV/H_2_. This effect may be partially due to the steric repulsion^47^ when the number of H_2_ molecules increases. In line with this trend, the Li–B distances are elongated gradually from 2.35 to 2.43 Å. However, the H–H bond distance is nearly constant in the range of 0.75–0.76 Å, which is the value of free H_2_ molecule, consistent with the nature of molecular adsorption. The Li–H distances span a rather wide range from 1.97 to 2.91 Å. Notably, there is an abrupt increase in the Li–H distances from **1**–3 H_2_ to **1**–4 H_2_, so that the first three H_2_ are closer to the Li site than the next three. In other words, the adsorption of the first three H_2_ molecules forms an inner core with Li, upon which the additional H_2_ molecules adsorb loosely. The data of consecutive AE confirm this to be indeed the case: The first three H_2_ possess an AE of 0.25–0.11 eV, in contrast to 0.04–0.05 eV for the next three (Table S1). In fact, the structure of **1**–4 H_2_ can be constructed on the basis of **1**–3 H_2_ by adding one H_2_ on the top of Li. However, when the fifth and sixth H_2_ are put on successively in **1**–5 H_2_ and **1**–6 H_2_, they flee away after structural optimization as shown in Fig. 5a. Therefore, the Li site in **1** may adsorb three H_2_ molecules comfortably, whereas additional H_2_ are only physisorbed.

Basically, the adsorption of H_2_ on **2** is rather similar to that on **1**. Up to five H_2_ molecules may be adsorbed on the Li site in **2** (Fig. 5b). Again, the first three H_2_ are located closely to Li, with the fourth H_2_ being situated symmetrically on top of Li at a substantially larger distance. For the **2**–5 H_2_ case, there is a structural rearrangement for the H_2_ molecules, suggesting that this Li site can potentially adsorb up to four H_2_ molecules at a reasonable strength. Nonetheless, the fifth H_2_ only interact with Li loosely. On the basis of the consecutive AEs (0.22–0.17 eV for the first three H_2_; Table S1), we conclude that the Li site in **2** is capable of adsorbing at least three H_2_ molecules with further possibility for a fourth, whereas additional H_2_ molecules should be considered physisorbed.

**Figure 6:**
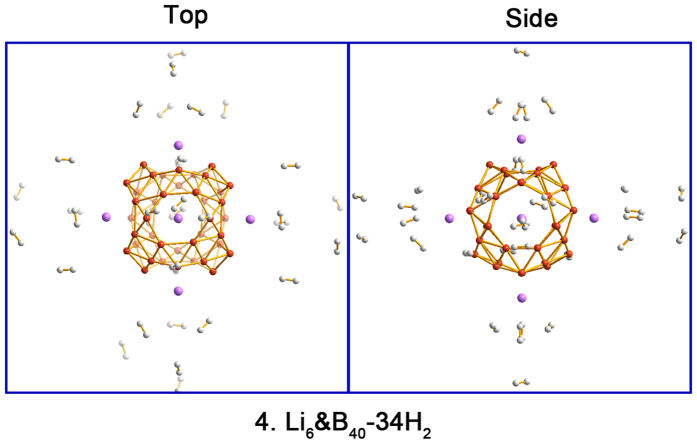
Top and side views of the optimized configuration of Li_6_&B_40_-34 H_2_(4). The B atom is in orange, Li in purple, and H in white.

Based on the above results of H_2_ adsorption on single Li-decorated B_40_, we constructed and optimized the H_2_ adsorption configurations on the Li_6_&B_40_(**3**) complex, which aims at exploring the hydrogen storage capacity. The starting configurations were constructed by attaching the corresponding H_2_ molecules around Li atoms above the 4 heptagonal and 2 hexagonal holes on the B_40_ cage. Successively, 6 H_2_, 12 H_2_, 18 H_2_, 24 H_2_, and up to 34 H_2_ are adsorbed on Li_6_&B_40_(**3**), whose optimized structures are shown in Fig. S2 and 6. The former four cases correspond to the adsorption of one to four H_2_ on each Li site. For the 34 H_2_ case, that is, Li_6_&B_40_-34 H_2_(**4**), 6 H_2_ are adsorbed on each heptagonal Li site and 5 H_2_ are on each hexagonal Li site, as depicted in Fig. 6. The total interaction energy of 34 H_2_ in **4** is 3.43 eV, yielding an average AE of 0.10 eV/H_2_. The calculated consecutive AEs are collected in Table 2, which reflects the adsorption nature more faithfully. Similar to **1** and **2**, the first three H_2_ for each Li in **4** are located close to the adsorption site, resulting in the **3**–6 H_2_, **3**–12 H_2_, and **3**–18 H_2_ complexes with reasonable AEs of 0.18–0.11 eV/H_2_. Additional H_2_ molecules in **3**–24 H_2_ and **4** complexes are farther away for the Li sites with relatively weak AEs of 0.03–0.05 eV/H_2_, hinting physisorption in nature. In summary, up to 34 H_2_ molecules may be adsorbed in **4**, among which the first 18 interact in moderate strength with the Li site, corresponding to a gravimetric density of 7.1 wt%.

### H_2_ Adsorption on Li-Decorated B_40_H_16_

**Figure 7:**
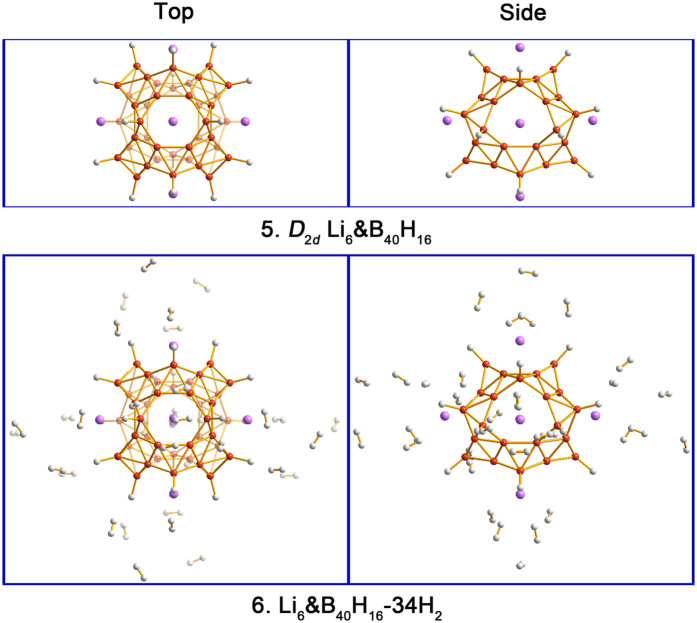
Top and side views of the optimized configurations of Li_6_&B_40_H_16_(5) and Li_6_&B_40_H_16_-34 H_2_(6). The B atom is in orange, Li in purple, and H in white.

Borospherene B_40_ as an electron-deficient system^36^ is generally considered to be more reactive than C_60_, we can thus engineer and passivate B_40_ at least partially with B–H bonds, which may benefit the adsorption and release of H_2_ molecules. B_40_ has 16 tetracoordinate and 24 pentacoordinate sites, where the former are anticipated to be more reactive. A model B_40_H_16_ cage cluster is readily constructed via 16 B–H bonds for the tetracoordinate B sites, which can also be decorated with six Li atoms, resulting in a *D*_2*d*_ Li_6_&B_40_H_16_(**5**) complex as depicted in Fig. 7. The Li–B distance in **5** remains to be 2.33 Å, which is very close to that in **3**. In complex **5**, each Li atom carries a charge of 0.88 |e|. Interestingly, the B–H bonds markedly alter the Li-decoration properties in **5** and the average BE of Li atom now increases to 4.17 eV per Li, compared to 3.07 eV in Li_6_&B_40_(**3**).

Li_6_&B_40_H_16_(**5**) can also adsorb from 6 H_2_, 12 H_2_, 18 H_2_, 24 H_2_, and up to 34 H_2_ molecules, resulting in a series of **5**-*n*H_2_ complexes (Fig. S3). The optimized structure for Li_6_&B_40_H_16_-34 H_2_(**6**) is shown in Fig. 7. Note that hydrogen remains in the molecular state with a uniform H–H distance of 0.75 Å in all **5**-*n*H_2_ species. For the first 6 H_2_ molecules in **5**–6 H_2_, the average AE amounts to 0.22 eV/H_2_. The average Li–B and Li–H distances, 2.33 and 1.97 Å, respectively, are almost the same as those in Li_6_&B_40_-6 H_2_ (that is, **3**–6 H_2_). With further H_2_ adsorption, the average AEs for the first 18 H_2_ in **5**-*n*H_2_ decrease slightly down to 0.17 eV/H_2_, which are in the ideal thermodynamic range for reversible hydrogen storage^10^,^11^. The Li_6_&B_40_H_16_(**5**) complex thus behaves rather similar to Li_6_&B_40_(**3**) in terms of hydrogen storage properties, except for the B–H passivation in **5**. The 18 “core” H_2_ in Li_6_&B_40_H_16_-34 H_2_(**6**) represents a gravimetric density of 6.5 wt%, where an additional 8.6 wt% of dissociated H atoms and loosely physisorbed 16 H_2_ are not counted.

### On the Possibility of Doubling the H_2_ Adsorption Sites: Li-Decorated Triangular B_6_ Corners

To further improve the hydrogen storage capacity of Li-decorated B_40_, we also attempted to place Li atoms on top of the close-packing, triangular B_6_ corner sites of the cube-like B_40_ cage. As a test case, adsorption of a single H_2_ molecule on a corner Li site is optimized (Fig. S4). The BE of Li is 1.87 eV, which is lower than those in Li&B_40_(**1**) and Li&B_40_(**2**), but the value still represents a reasonable strength. In fact, it is comparable to the corresponding value for C_60_ (1.80 eV)^13^. Moreover, the AE for the first H_2_ amounts to 0.28 eV, which is comparable to and even slightly greater than that in Li&B_40_(**1**)-H_2_ (0.25 eV) or Li&B_40_(**2**)-H_2_ (0.22 eV; Table S1). The above data hint that a triangular B_6_ corner site on B_40_ is as promising as, if not better than, a hexagonal/heptagonal site for hydrogen storage. The calculated consecutive AEs of Li&B_40_-nH_2_ (n = 1–3) with H_2_ molecules adsorbed on a corner Li site are at the range of 0.22–0.28 eV/H_2_. For Li_8_&B_40_-nH_2_ (n = 8, 16, 24) with H_2_ molecules adsorbed on eight corner Li site, the consecutive AEs change from 0.17 to 0.36 eV/H_2_, which are ideal for reversible hydrogen storage and release

**Figure 8:**
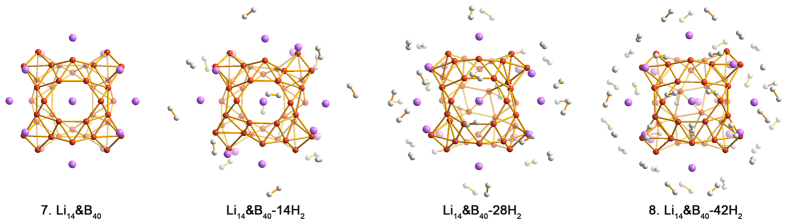
Optimized configurations of Li_14_&B_40_(7) and its H_2_ adsorption complexes: Li_14_&B_40_-14 H_2_, Li_14_&B_40_-28 H_2_, and Li_14_&B_40_-42 H_2_(8). The B atom is in orange, Li in purple, and H in white.

In this way, one can more than double the number of sites for Li-decoration from six in Li_6_&B_40_(**3**) and Li_6_&B_40_H_16_(**5**) to fourteen in Li_14_&B_40_(**7**), owing to the eight triangular B_6_ corners (versus six hexagonal/heptagonal holes). The optimized structure of Li_14_&B_40_(**7**) is shown in Fig. 8. Here, upon Li decoration, the boron structure distorts considerably from the free-standing B_40_ borospherene, but the cage motif maintains. The average BE is 2.57 eV/Li. Following the strategy for Li_6_&B_40_(**3**) and Li_6_&B_40_H_16_(**5**), we build a series of model complexes: **7**–14 H_2_, **7**–28 H_2_, and Li_14_&B_40_-42 H_2_(**8**), whose optimized structures are depicted in Fig. 8. The calculated average AE for the first 14 H_2_ in **7**–14 H_2_ is 0.32 eV/H_2_, for the second 14 H_2_ in **7**–28 H_2_ is 0.22 eV/H_2_, and for the third 14 H_2_ in Li_14_&B_40_-42 H_2_(**8**) is 0.12 eV/H_2_, suggesting that all these H_2_ molecules are thermodynamically favorable for a hydrogen storage material^10^,^11^. For the extreme case of **8**, a maximum gravimetric density of 13.8 wt% is obtained. We do not exclude the possibility of further H_2_ adsorption onto the **8** complex, albeit those additional H_2_ are anticipated to interact rather loosely with the Li sites.

## Concluding Remarks

In conclusion, we have carried out a comprehensive density-functional study on the lithium-decoration of B_40_ borospherene and the potential utilization of Li-B_40_ complexes as a novel nanomaterial for hydrogen storage. We showed that all six heptagonal and hexagonal holes on B_40_ surface can be decorated with Li atoms and each Li site is capable of adsorbing up to six or five H_2_ molecules. This results in an ultimate Li_6_&B_40_-34 H_2_ complex, in which 18 H_2_ are bound to Li sites with ideal adsorption energies of 0.11–0.18 eV per H_2_, corresponding to a gravimetric density of 7.1 wt%. The additional 16 H_2_ are physisorbed in nature. We further showed that the eight close-packing, triangular B_6_ corner sites on the B_40_ cage are also readily decorated with Li, which more than double the number of sites for hydrogen storage. The corresponding Li_14_&B_40_-42 H_2_ complex can store all 42 H_2_ molecules at adsorption energies of 0.12–0.32 eV per H_2_, suggesting a maximum gravimetric density of 13.8 wt%. The Li-B_40_-H_2_ complexes as a hydrogen storage material differ markedly from the prior Li-C_60_-H_2_ and Ti-B_40_-H_2_ systems. The Li-C_60_-H_2_ complex^13^ adsorbs H_2_ rather loosely and is thus not efficient for hydrogen storage, whereas the Ti-B_40_-H_2_ complex^40^ bounds H_2_ too strongly, for which a substantial portion of H_2_ stored are not reversible for release. In fact, preliminary attempts also suggest that the structural integrity of B_40_ unit is maintained when they are allowed to interact with each other. Considering the presence of chemical bondings between them, we forecast it is possible to construct boron-based nanomaterials for hydrogen storage using lithium-decorated B_40_ unit as a building block or connecting the exohedral metalloborospherene with organic linkers. And the hydrogen storage capacity of the boron-based nanomaterials could be better than previously reported carbon-based counterparts.

## Methods

All calculations were based on DFT, using a plane-wave basis set with the Projector Augmented Wave (PAW)^48^,^49^ pseudopotential method as implemented in the Vienna *ab initio* Simulation Package (VASP)^50^,^51^. Generalized gradient approximation (GGA) with the Perdew-Burke-Ernzerhof (PBE)^52^ functional was adopted to treat the electron exchange correlation. The GGA-PBE method has been previously utilized to treat Li-decorated fullerenes and heterofullerenes for hydrogen storage^19^,^53^ which is thus a suitable choice for our current system. The dispersion corrected DFT (DFT-D) scheme^54^,^55^,^56^ was used to describe the van der Waals (vdW) interaction. The supercell approach was used, where the B_40_-based systems were placed at the center of a 25 × 25 × 25 Å^3^ vacuum space. And only the Γ point was used to sample the Brillouin zone. The energy cutoff for the plane-wave basis set was set to 500 eV. All structures were fully relaxed until the force acting on each atom was less than 10^−2 ^eV/Å and a tolerance in total energy was at least 10^−4 ^eV.

The binding energies (BEs) for the Li-decorated B_40_ are defined as E_b_ = −(E_Li-B40_ −E_B40_ − *m*E_Li_)/*m*, where E_Li-B40_ is the total energy of Li-decorated B_40_, E_B40_ and E_Li_ are the total energies of an isolated B_40_ and a Li atom, respectively, and *m* is the number of Li atoms. Similarly, the average AE for H_2_ is defined as E_a_ = −(E_Li-B40-*n*H2_ − E_Li-B40_ − *n*E_H2_)/*n* and the consecutive AE is defined as ΔE = −(E_Li-B40-*n*H2_ − E_Li-B40-(*n*-1)H2_ − E_H2_), where E_Li-B40-*n*H2_ and E_Li-B40-(*n*-1)H2_ are the total energies of *n* and (*n*–1) H_2_ adsorbed on the Li-decorated B_40_, respectively. E_Li-B40_ also represents the total energy of Li-decorated B_40_, E_H2_ is the total energy of isolated H_2_ molecule, and *n* stands for the number of adsorbed H_2_ molecules.

We note that for comparison with *D*_2*d*_ B_40_ in our previous work (ref. 36), the HOMO-LUMO energy gaps of **1**, **2**, and **3** were calculated using the Gaussian 09 package^57^, which is usually used for calculations on the isolated molecules. And the corresponding structures were optimized at the PBE0 levels with the 6–311 + G* basis set^58^,^59^, which has been benchmarked in prior works as a reliable method for boron clusters.

## Additional Information

**How to cite this article**: Bai, H. *et al*. Lithium-Decorated Borospherene B_40_: A Promising Hydrogen Storage Medium. *Sci. Rep.* **6**, 35518; doi: 10.1038/srep35518 (2016).

## Supplementary Material

Supplementary Information

## Figures and Tables

**Table 1:** Calculated Binding Energy (BE) of Li to B_40_, Li–B Bond Distance, and Atomic Charge on Li Atom for Li&B_40_(**1**), Li&B_40_(**2**), and Li_6_&B_40_(**3**).

system	BE/Li (eV)	R_Li-B_ (Å)	charge on Li (|e|)
*C*_*s*_ Li&B_40_(**1**)	3.08	2.34	0.88
*C*_2*v*_ Li&B_40_(**2**)	2.88	2.33	0.87
*D*_2*d*_ Li_6_&B_40_(**3**)	3.07	2.33	0.87

**Table 2:** Calculated Average and Consecutive Adsorption Energy (AE) of H_2_, Bond Distances of Li–B, Li–H, and H–H in the Li_6_&B_40_-*n*H_2_ (*n* = 6, 12, 18, 24, 34) Complexes.

system	AE/H_2_(eV) (average)	AE/H_2_ (eV) (consecutive)	R_Li-B_ (Å)	R_Li-H_ (Å)	R_H-H_ (Å)
Li_6_&B_40_-6 H_2_	0.18	0.18	2.34	1.99	0.75
Li_6_&B_40_-12 H_2_	0.17	0.17	2.37	2.00	0.76
Li_6_&B_40_-18 H_2_	0.15	0.11	2.41	2.08	0.76
Li_6_&B_40_-24 H_2_	0.12	0.03	2.41	2.54	0.76
Li_6_&B_40_-34 H_2_	0.10	0.05	2.42	2.94	0.76
