# Supplementary material for: Lithium-Decorated Borospherene B40: A Promising Hydrogen Storage Medium
Source: Sci Rep. 2016 Oct 18;6:35518. doi: 10.1038/srep35518 (PMC5067665; doi:10.1038/srep35518)
Supplement: Supplementary Information [file srep35518-s1.pdf]

SUPPORTING INFORMATION

Lithium-Decorated Borospherene B_{40}: A Promising Hydrogen Storage Medium

Hui Bai,[†] Bing Bai,[†] Lin Zhang,[†] Wei Huang,*[,†] Yue-Wen Mu,[‡] Hua-Jin Zhai,*[,‡,§] & Si-Dian Li[‡]

[†]*Key Laboratory of Coal Science and Technology of Ministry of Education and Shanxi Province, Taiyuan University of Technology, Taiyuan 030024, Shanxi, China*

[‡]*Nanocluster Laboratory, Institute of Molecular Science, Shanxi University, Taiyuan 030006, Shanxi, China*

[§]*State Key Laboratory of Quantum Optics and Quantum Optics Devices, Shanxi University, Taiyuan 030006, Shanxi, China*

*E-mail: huangwei@tyut.edu.cn; hj.zhai@sxu.edu.cn

Table S1. Calculated average and consecutive adsorption energy of H_2, bond distances of Li–B, Li–H, and H–H in the Li&B_{40}(**1**)-nH_2 and Li&B_{40}(**2**)-nH_2 (n = 1–6) complexes.

system	AE/H_2 (eV) (average)	AE/H_2 (eV) (consecutive)	R_{Li-B} (Å)	R_{Li-H} (Å)	R_{H-H} (Å)
Li&B_{40} (**1**)-H_2	0.25	0.25	2.35	1.97	0.76
Li&B_{40} (**1**)-2H_2	0.20	0.15	2.38	2.02	0.76
Li&B_{40} (**1**)-3H_2	0.17	0.11	2.42	2.06	0.75
Li&B_{40} (**1**)-4H_2	0.14	0.04	2.42	2.53	0.75
Li&B_{40} (**1**)-5H_2	0.12	0.05	2.43	2.76	0.75
Li&B_{40} (**1**)-6H_2	0.11	0.05	2.43	2.91	0.75
Li&B_{40} (**2**)-H_2	0.22	0.22	2.34	1.97	0.76
Li&B_{40} (**2**)-2H_2	0.19	0.17	2.38	2.02	0.76
Li&B_{40} (**2**)-3H_2	0.19	0.17	2.41	2.02	0.76
Li&B_{40} (**2**)-4H_2	0.15	0.04	2.41	2.48	0.76
Li&B_{40} (**2**)-5H_2	0.12	0.01	2.42	2.50	0.76

Figure S1. Two isomers of Li_6B_{40} with a Li_6 cluster attaching on top of a heptagonal/ hexagonal hole of B_{40}.

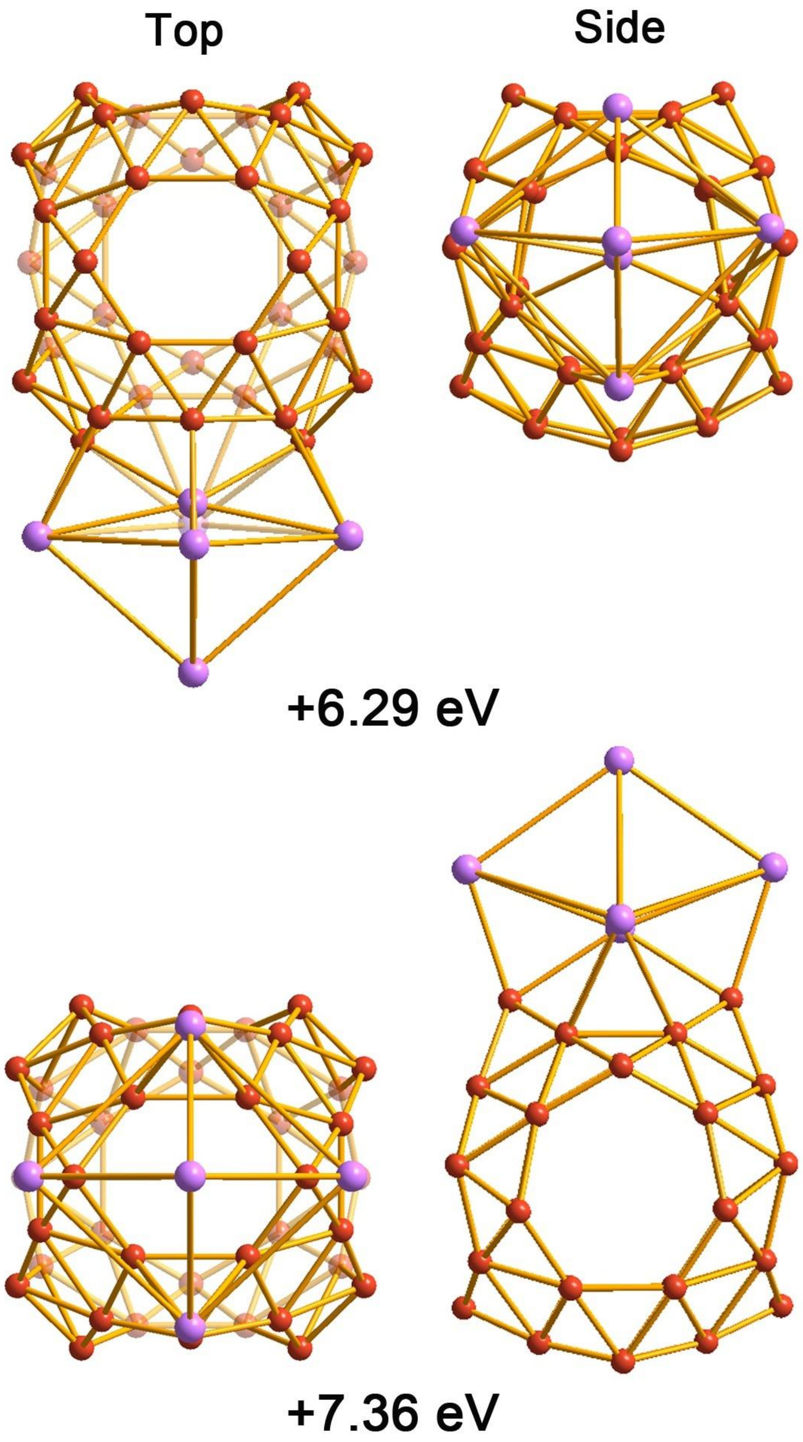

Figure S2. Top and side views of optimized configurations of successive addition of H_2 molecules on $Li_6\&B_{40}$(**3**).

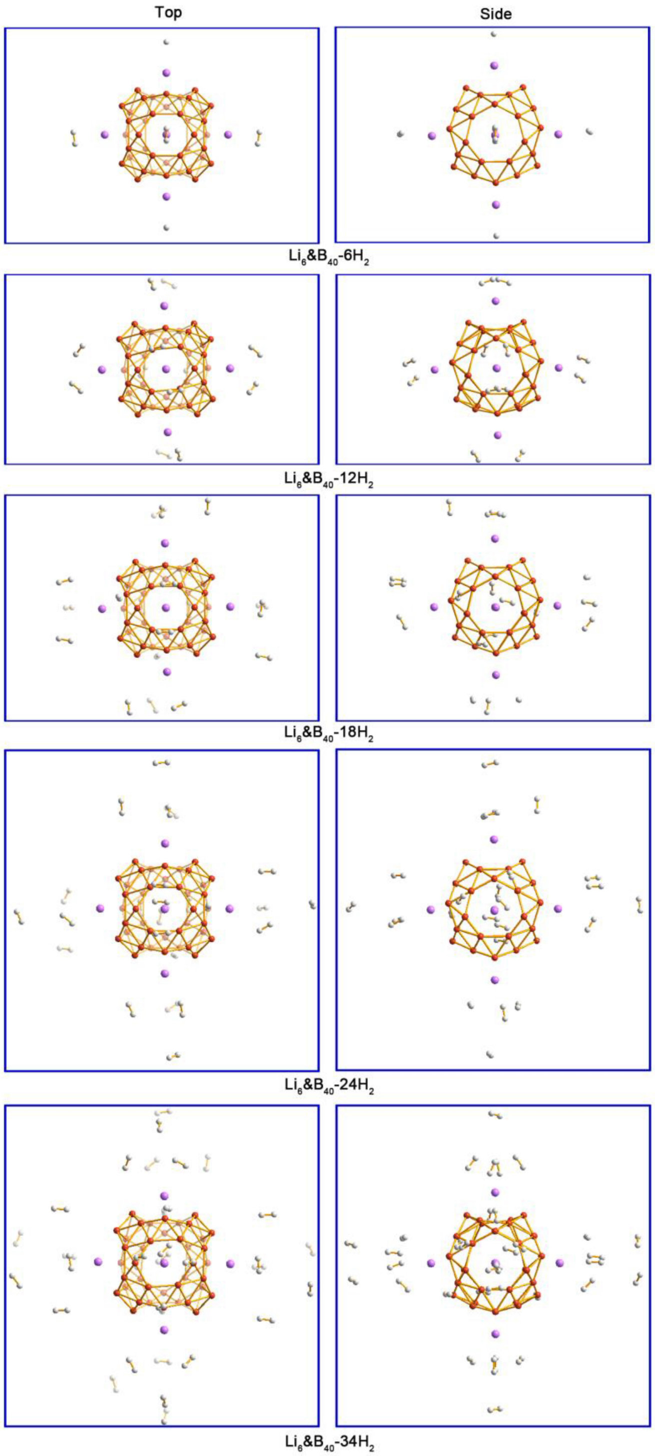

Figure S3. Top and side views of optimized configurations of successive addition of H_2 molecules on $Li_6\&B_{40}H_{16}$(**5**).

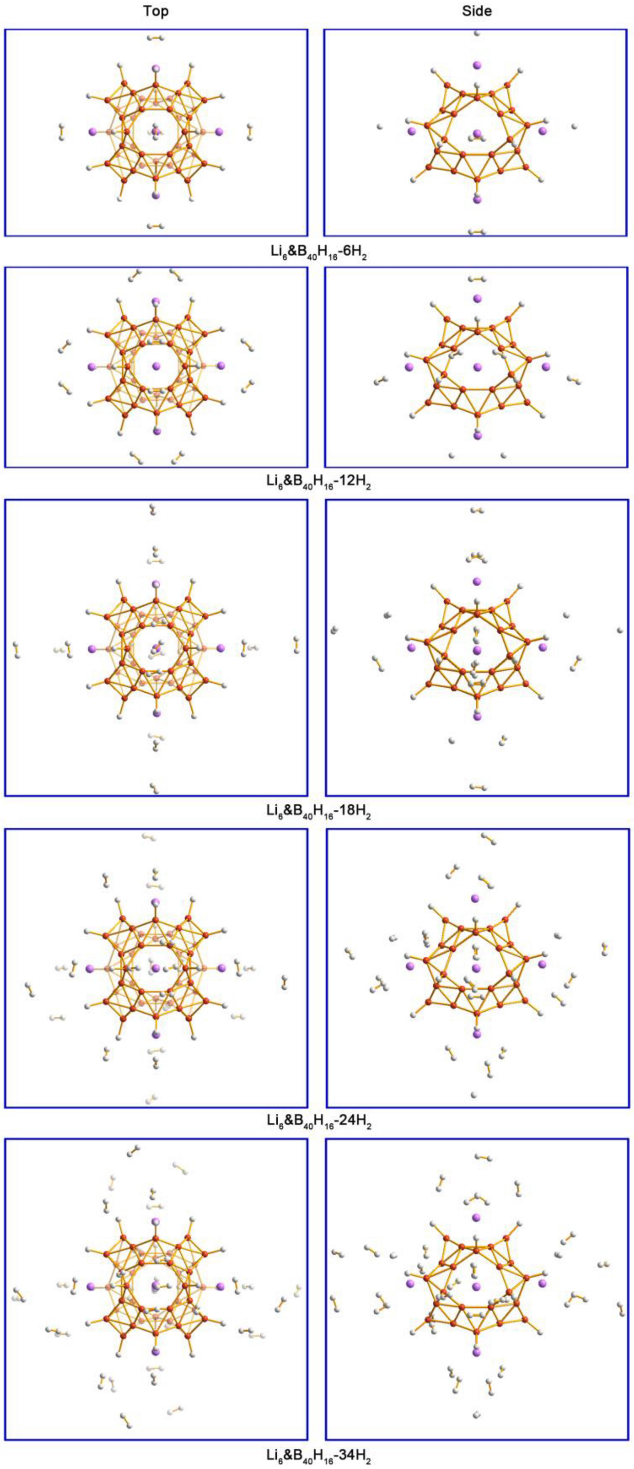

Figure S4. Optimized configuration of Li&B_{40}-H_2, where a Li is attached on a close-packing B_6 corner site of B_{40}.

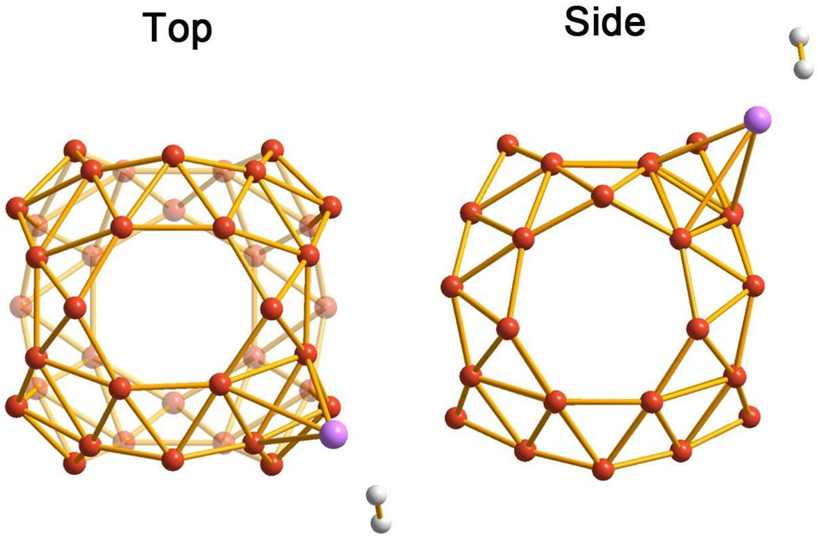

Li&B_{40}-H_2
